# Moral Challenges in Transgender Care: A Thematic Analysis Based on a Focused Ethnography

**DOI:** 10.1007/s10508-018-1287-3

**Published:** 2018-09-18

**Authors:** Karl Gerritse, Laura Hartman, Marte Fleur Antonides, Annelijn Wensing-Kruger, Annelou L. C. de Vries, Bert C. Molewijk

**Affiliations:** 1 Department of Medical Humanities, VU University Medical Centre, Amsterdam Public Health, De Boelelaan 1089a, 1081 HV Amsterdam, The Netherlands; 2 Center of Expertise on Gender Dysphoria, VU University Medical Center, Amsterdam, The Netherlands; 3 Faculty of Medicine, Center for Medical Ethics, Institute of Health and Society, University of Oslo, Oslo, Norway

**Keywords:** Gender dysphoria, Transgender, Moral challenges, Ethical challenges, Ethics support, DSM-5

## Abstract

Treatment teams providing transgender-affirming medical care are inherently faced with various kinds of moral and ethical dilemmas and questions, which are becoming even more pressing due to increasing treatment numbers and public attention for transgender care. Little is known about what kinds of moral and ethical challenges manifest in clinical practice. The aim of the present research was to map the moral and ethical challenges of healthcare professionals working in a specialized multidisciplinary transgender care center. Over a period of 7 months, during a focused ethnographic study, data were collected through participant observation of multidisciplinary team meetings, observation of individual psychodiagnostic assessment sessions with clients, and analysis of transcripts and reports of a series of moral case deliberations. A thematic content analysis of the data identified various implicit and explicit moral and ethical challenges around the following six themes: (1) assessing eligibility; (2) content of treatment; (3) sequential order of the treatment steps; (4) role of the clinical guidelines; (5) differing notions regarding gender identity, and (6) decision-making process. Our research provides a detailed insight into the way healthcare professionals experience these moral and ethical challenges and how they are related to (local) guidelines, the multidisciplinary character of GD care, and its inherent implicit and explicit gender norms. Our findings suggest that good transgender care may profit from continuous multidisciplinary deliberation of and sensitivity toward the normative dimension of transgender care. The paper ends with recommendations for ethics support mechanisms in transgender care.

## Introduction

In countries where healthcare professionals use the *Diagnostic and Statistical Manual of Mental Disorders* (DSM), those who experience distress due to incongruence between one’s experienced gender identity and sex assigned at birth may meet the criteria for a diagnosis of gender dysphoria (GD) (American Psychiatric Association, 2013). Transgender-affirming treatment of gender dysphoria is available to aid individuals in exploring their gender identity and coping with their distress, with the aim to listen to and unravel together what one is conveying and feeling about gender identity and gender expression (Hidalgo et al., 2013). Sometimes, transgender-affirming treatment includes bodily alterations to aid a client in expressing their experienced gender: Sequentially, medical care options include hormonal puberty suppression (in youth), feminization or masculinization through hormone therapy and/or surgery (Coleman et al., 2012).

The practice of diagnosing and treating GD is complex (Drescher & Byne, 2012; Stein, 2012), and moral and ethical challenges are ubiquitous (Swann & Herbert, 2008). In line with Hartman, Metselaar, Widdershoven, and Molewijk (2018a), we delineate the concept of “morality” as a moral background consisting of an intricate web of norms, values, responsibilities and reserve the term “ethics” for the more actual discussion of or reflection on these values and norms (Dewey, 2002; Keulartz, Schermer, Korthals, & Swierstra, 2004). Moral challenges are here defined as situations in which the stakeholder who experiences the challenge is uncertain or in disagreement about what is morally right to do. An ethical challenge is seen as a challenge related to how to deal with or solve a moral challenge (see Molewijk, Hem, & Pedersen, 2015 for a typology of moral and ethical challenges). In the literature on gender-affirming medical care various moral and ethical challenges have been described ranging from theoretical and taxonomical to more pragmatic and clinical. The latter are challenges such as how to go about issues of assent in the case of transgender clients suffering from comorbidity such as autism (Shumer & Tishelman, 2015), or more implicit ones such as whether it is ethically permissible to offer puberty suppression to adolescents, given that its medical and psychosocial risks have not been fully established (Vrouenraets, Frederiks, Hannema, Cohen-Kettenis, & de Vries, 2016).

When providing care to clients, clinicians may draw from international guidelines and standards such as the World Professional Association for Transgender Health (WPATH) Standards of Care (SoC) (Coleman et al., 2012) and the Endocrine Society Guidelines (Hembree et al., 2017). These guidelines are purposefully flexible so as to meet a variety of transgender healthcare needs and service providers. In multidisciplinary transgender clinics, local guidelines are often devised, incorporating knowledge and considering (infrastructural, practical) expertise of the disciplines involved.

However, these guidelines are expert opinion consensus statements offering rough clinical guidelines in the absence of robust empirical research. The extent to which both international and local guidelines can aid clinicians in grappling with the contingencies from which moral and ethical challenges arise in the provision of care is equivocal and has been questioned. For example, cases involving parental apprehension toward transitioning (Bernal & Coolhart, 2012) or severe coexisting psychopathology (Vrouenraets, Frederiks, Hannema, Cohen-Kettenis, & de Vries, 2015) can leave clinicians morally divided on how to proceed with treatment. There is little explicit support for clinicians to cope with moral challenges in transgender-affirming medical care. A deliberative handling of these challenges may not only aid professionals in their practice, but also establish a reference to identify what “good” care is. Attention and sensitivity toward the latter is of importance, especially in the light of moral distress experienced by clinicians in facing opposing (multidisciplinary) values from colleagues or transgender clients (Thisthlethwaite & Hawksworth, 2015).

Against a backdrop of increasing numbers and decreasing age of referrals (Aitken et al., 2015; de Vries & Cohen-Kettenis, 2012), these moral challenges become even more pressing. Indeed, they have been the subject of various publications. Some authors made use of composite case narratives based on the authors’ experience working with the transgender community (Bernal & Coolhart, 2012; Swann & Herbert, 2008; Tishelman et al., 2015), others drew retrospectively from case histories without systematic data collection (Giordano, 2008; Wiseman & Davidson, 2012) or did not use empirical data to support the moral challenges they raised (Drescher & Pula, 2014; Pomora et al., 2015). However, moral challenges and themes are intricately linked to the context in which they arise and how they are experienced (Molewijk, Abma, Stolper, & Widdershoven, 2008). Particularly, studies investigating how moral challenges in transgender-affirming medical care manifest and are experienced by clinicians in the embedded context of everyday clinical practice appear to be missing.

The aim of this study is to systematically map the moral and ethical challenges experienced in everyday clinical practice by professionals working in a multidisciplinary transgender team (i.e., during team meetings, outpatient services and specific clinical ethics support sessions). To this end, a qualitative, focused ethnography (Cruz & Higginbottom, 2013) will be conducted allowing us to add to the current understanding of the moral and ethical dimensions of transgender care, by shifting the current focus to a more clinical and experiential one embedded in a distinct context. Specifically, this study incorporates both the experience of clinicians and observations of implicit moral challenges by the researchers to voice the moral and ethical deliberations of clinicians and enhance awareness of the embedded nature in which moral challenges in transgender-affirming medical care arise. In this sense, our overall research question was: What moral and ethical challenges are present in the diagnosis and treatment of individuals with gender dysphoria in a multidisciplinary transgender clinic in the Netherlands?

We emphasize that this descriptive study is a first step in revealing and making explicit these moral and ethical challenges from clinical practice. Based on our theoretical viewpoints on clinical ethics and integrative ethics support (Hartman et al., 2018a; Widdershoven, Abma, & Molewijk, 2009; Widdershoven & Molewijk, 2010) we did not intent to normatively settle the various moral and ethical challenges right away within this paper. Only after publishing these results, a second step can follow: starting an (international) dialogue and normatively reflecting upon the challenges and how they could be handled.

## Method

### Setting

In Amsterdam, transgender-affirming medical care is provided by the Center for Expertise and Care for Gender Dysphoria (CEGD). The CEGD’s guidelines largely follow the SoC of WPATH (Coleman et al., 2012), but are adapted to a local (infrastructural, professional, and legal) context. For example, while closely mimicking the eligibility criteria mentioned in the SoC, the local guidelines include health determinants such as body mass index (BMI), smoking and age requirements as eligibility criteria for, sequentially: puberty suppression, cross-hormones and surgery. The local guidelines delineate that a case-by-case decision is always possible.

From 2013 onward, the CEGD receives structural and integrated clinical ethical support (CES) from the medical humanities department of VU University Medical Center (VUmc). CES aims to support clinical practitioners to reflect upon the quality of care through elucidating their moral challenges and stimulating reflection and dialogue (Hartman et al., 2018a). At the CEGD, CES was introduced in the multidisciplinary team through the use of moral case deliberation (MCD) (Molewijk et al., 2008). To clarify, MCD is a facilitator-led collective moral inquiry by healthcare providers into a moral question connected to a real clinical case. In this study, MCD was used as a data collection method.

### Research Team

Data were collected during the winter of 2015–2016. The research team consisted of a medical and bioethics student (KG, MD), a clinical ethics researcher (LH, Ph.D. candidate), a child and adolescent psychiatrist (AdV, M.D. and Ph.D.), a medicine student (MFA, B.Sc.), a medical psychologist (AWK, M.Sc.), and an ethicist and senior researcher (BM, Associate Professor). Participant observations were carried out by KG (MCDs, multidisciplinary and individual consultations), LH and BM (multidisciplinary team meetings and both facilitators of MCDs). MFA helped during data analysis. AdV and AWK, part of a multidisciplinary transgender team, were both participant and researcher in this study.

### Study Design

This research combined various data collection methods (see Table 1 for an overview): (1) observation of multidisciplinary team meetings. In these meetings, difficult and (potentially morally or ethically) troublesome treatment decisions are discussed and made. During these meetings, psychologists, psychiatrists, endocrinologists, pediatricians, and plastic surgeons are present in alternation and treatment decisions are made, based on consensus. KG carried out 10 observations (5 children/adolescent and 5 adult team meetings), at which LH and BM were present at four of these 10 observations. During the observations, detailed field notes were taken on (moral and ethical) content and questions that arose to the researchers. (2) Observation of individual psychodiagnostic sessions between clinicians and transgender individuals (10x). Preliminary themes were identified and used to determine a purposeful selection for the observation of individual consultations. (3) Moral case deliberation. This research made use of analyses of (1) transcripts of MCD (7 ×); (2) reports of MCD (22 ×), written by the facilitator and member checked by the MCD participants; (3) field notes taken during MCD (4 ×). During the time of data collection, LH, BM and a colleague (physician) facilitated and KG observed 4 MCDs. Field notes and written reports were made of all four MCD’s, and three out of four MCDs were audio-recorded, transcribed verbatim (duration around 90 min). The remainder of MCD data is derived from a dataset used in a larger study context into the role of MCD in dealing with moral dilemmas in transgender care by LH and BM.

**Table 1:** Overview of the dataset

Data collection method	Type of data	Amount	Selection
Moral case deliberations	Transcripts	7 ×	Convenience sample
	Reports	22 ×	Convenience sample
	Field notes	4 ×	Convenience sample
Multidisciplinary meetings	Field notes	12 ×	Convenience sample
Individual consultations	Field notes	10 ×	Purposive sample

### Analysis

The data were analyzed using a thematic content analysis (Green & Thorogood, 2014), a systematic method to map content and topics across a dataset. The data were inductively analyzed in MAXQDA 12.0 by means of open codes, which entails that all potentially relevant textual fragments were coded by the researchers, without hierarchy between the types of data.

Chronologically, the coding process took place as follows. The first author (KG) inductively coded all data and compared codes with the third author (MFA) who coded 2 MCDs, 2 interviews and 4 observational reports. The latter author was hitherto not involved with the team and unfamiliar with the type of care and thus offered an independent perspective. This resulted in an initial code list. These two authors discussed codes in an open manner, i.e., they did not yet identify (sub)themes, and reached consensus/resolved discrepancies by discussing and determining which code fitted best the content of a particular textual fragment.

Next, the first (KG), second (LH) and sixth (BM) authors independently coded one transcript, compared and discussed the codes, after which they developed a coding tree. This consisted of codes–subcodes–segments with a corresponding codebook defining the various codes. The first author (KG) then applied this coding tree to the field notes, with new codes being added to the coding tree. The first author (KG) also applied it to the data analyzed earlier, by regularly checking and rephrasing specific codes, by developing new hierarchies and by discussing it until reaching consensus with the second (LH) and sixth author (BM).

During the third phase, the first (KG), second (LH), and sixth (BM) authors reconvened to discuss and reach consensus on the initial themes that emerged from the hierarchical coding tree. They grouped the codes (based on the textual fragments) together based on the emerging themes and subthemes, while checking for interpreter consensus concerning this assignment.

This procedure resulted in the identification of six themes of moral challenges. In our analysis, we identified both explicit and implicit moral and ethical challenges and an overarching moral question for every main theme that summarizes the moral content within it. Explicit moral and ethical challenges are those experienced and verbalized by the healthcare professionals, whereas implicit ones remain largely under-discussed and are made explicit through interpretations of the authors. In our study, through identifying implicit moral and ethical challenges and confronting the team members with these implicit challenges, e.g., during the member check, we sought to bring about—in a non-directive manner—a moral reflection onto certain themes and challenges that were potentially overlooked by members of the team. In doing this, we accepted that what some consider a moral or ethical challenge might not be seen or experienced as such by others: This is in keeping with our theoretical background that these challenges do not exist in and of themselves.

This research is part of a movement within clinical ethics support (CES) that views experience as the source of morality (Abma, Baur, Molewijk, & Widdershoven, 2010; Widdershoven et al., 2009; Widdershoven & Molewijk, 2010) and stresses the value of fostering ethical reflection together with caretakers in the context of daily practice, rather than a detached moral or ethical reflection or judgment. In this light, the experience, moral questions and expertise of caretakers, rather than theoretical knowledge of ethics, are prioritized. Our role as researchers was then not to (pre)determine what is or is not moral or ethical, but rather to identify, extract and describe the moral and ethical dimensions experienced by the team members within the moral case discussions we led and team meetings and individual meetings we observed (i.e., when people started to question their moral or ethical background, either implicitly or explicitly).

### Quality Procedures

In qualitative research, data saturation usually dictates sample size (Cruz & Higginbottom, 2013). Due to practical considerations, in this research, a set amount of observations took place. Data saturation was reached after the observation and analysis of MCDs and multidisciplinary observations. However, it was not reached in the individual consultations because of the heterogeneity of the clinician–client dyads. A member check with the participating clinicians of the CEGD took place in the form of a presentation and discussion of a synthesis of the data and provisionary results. The analysis and findings reported below are based on a triangulation (Mays & Pope, 2000) of three types of data (transcripts and reports of MCD, detailed field notes on multidisciplinary and individual meetings).

### Ethical Considerations

The study was submitted for review to an officially accredited IRB/REC, the Medical Ethics Committee of the VUmc, which issued a declaration that under Dutch law full ethical review was not necessary (IRB00002991, January 21, 2016). The management team, partaking clinicians and those present during individual consultations gave their oral informed consent for the research to be carried out. All participants in the study were informed that participation in the study was voluntary and that they could withdraw from the study at any moment, without providing motivation. Participant anonymity is preserved in this writing. Some characteristics in quotes/observations have been altered slightly to attain the latter. In addition, in order to safeguard sensitivities and vulnerabilities of the multidisciplinary team, some primary expressions and responses by caretakers have been edited or omitted from the manuscript, without changing the specific moral theme or content at hand.

## Results

Six main themes of moral challenges in transgender-affirming medical care were identified in the data (see Table 2): (1) assessing eligibility; (2) content of treatment; (3) sequential order of the treatment steps; (4) role of the guidelines; (5) notions regarding gender identity/GD, and (6) decision-making process. The themes and their subthemes will be discussed below, illustrated and substantiated by quotes and field notes from the data. Rather than providing an all-including overview of the dataset, the illustrations serve as a means to demonstrate the morally troublesome nature of the themes in clinical practice and are selected based on methodological clarity and ethical significance. It is important to note that the quotes from individual employees and observations do not necessarily reflect the views of the CEGD.

**Table 2:** Frequency and percentages of coded segments per (sub)theme

Theme	Frequency	Percentage (%)
Assessing eligibility	415	26.0
Content of treatment	222	13.9
Sequential order of the treatment steps	154	9.6
Role of the (local) guidelines	144	9.0
Notions regarding gender identity/GD	175	11.0
Decision-making process	486	30.5

### Assessing Eligibility

The process through which (potentially) gender dysphoric individuals are assessed for transgender-affirming medical treatment is intricate. We identified four subthemes leading to moral and ethical challenges: (1) determining distress in order to diagnose GD, (2) overseeing the consequences of treatment, (3) estimating one’s coping with the effects of treatment, and (4) the influence of health determinants. The overarching moral question within this theme is: Under what circumstances ought transgender individuals to be rendered ineligible for transgender-affirming medical treatment?

#### How Should We Go About Determining Distress?

At the CEGD, in order to commence treatment, one has to meet the criteria of a formal DSM diagnosis (American Psychiatric Association, 2013). An essential criterion is the existence of clinically significant distress, i.e., distress related to a marked incongruence between one’s experienced/expressed gender and primary and/or secondary sex characteristics leading to significant problems functioning. However, some children and adolescents under discussion were showing only minimal suffering, causing a divergence between eligibility for medical treatment and a formal diagnosis which proved to be ethically difficult for some members of the multidisciplinary team:

And at the same time, what is relatively odd is that there hasn’t been any distress. … Based on this story, we can’t really diagnose, but I do feel that treatment is indicated, so it’s complicated. (transcript MCD)

This quote above raises the following moral question: Should we treat children and adolescents even though they show little distress?

#### Should the Client Envision the Consequences of Treatment Before We Initiate Treatment?

The hormonal treatment for GD is usually a life-long commitment and only partially reversible, while the surgical treatment is fully irreversible. The members of the multidisciplinary team found it important to assess whether those opting for treatment were able to envision the consequences (i.e., medical risks/side effects) of these treatment options. Sometimes, when someone had a low IQ, suffered from psychiatric comorbidity, or was young, this raised doubt: “Can she envision the consequences of a decision that will have a span of multiple years?” (transcript MCD). Consequently, a moral question for some of the team members is whether one should start treatment when its implications were not fully understood, e.g., in this case involving a 17-year-old trans boy:

Participant 1: That IQ test makes it problematic. What is our responsibility in making this treatment decision when he’s intellectually disabled? Then we should make it, right? Participant 2: Are we not thinking too much for the boy? Intellectually disabled or not, he should be able to choose. Participant 3: Well, then he should be able to understand what he chooses. (transcript MCD)

In the quotes above, and more generally during observations of team meetings dealing with psychiatric comorbidity such as autism, caretakers were weighing respect for autonomy with a good enough informed consent regarding the consequences of treatment.

#### Should We Know for Sure that Somebody Can Cope with Treatment Before We Start It?

We observed three criteria that were colloquially used by clinicians to determine whether someone is expected to cope with the effects of treatment: having a stable home situation, personal resiliency and a successful social transition. However, the use of these criteria led to moral and ethical questions in the team.

Regarding the criterion of personal resiliency, one team member expressed apprehension discussing a client who showed dysfunctional coping skills in other life domains. The clinician was unsure whether her client would be resilient enough to cope with the (side)effects of transgender-affirming medical treatment or in stead benefit from starting the latter after having gone through psychological training to enhance her coping skills. During a moral case deliberation, she expressed the following: “Will she be resilient enough when complications occur? I’m worried about the psychic suffering” (transcript MCD) When made more explicit, the following ethical question may be distilled from this case: In seeking to offer “good care”, should we offer someone to start treatment if we are not completely sure whether that person can cope with the (side)effects of treatment? Relatedly, what consequences may a clinician draw from their vision on the latter?

Regarding the third criterion, that of a successful social transition, the following case was discussed during a team meeting. In this particular case, some family members did not accept nor acknowledge the transgender identity and expression of their transgender sister and daughter. This resulted in a state in which she expressed her experienced gender to some, but not all people in her social environment. To some team members, one’s ability to cope with a social transition is an indication to what extent one might “cope” with (hormonal, surgical) treatment and hence informally sometimes used as one of the means to gauge eligibility for treatment. This case laid bare that it was not always clear what comprises a social transition. One of the clinicians present at that meeting asked: “What does that actually entail, a full social transition?” A discussion ensued regarding what a “proper” social transition might be comprised of (i.e., being open to all family members, or just a few)? In effect, this ambiguity problematized the notion that a successful social transition can be used as an indicator that one can “cope” with treatment.

#### When Should Health Determinants Become Exclusion Criteria?

Clinicians struggled with weighing the role and function of substance abuse or height of BMI against potential medical downsides. At the CEGD, these health determinants are included in the local clinical guidelines. For example, a high BMI is an exclusion factor for certain surgical options, in order to prevent or minimize the chances of surgical complications occurring. Determined by the risks involved in a particular type surgery, the BMI criterion is more lenient for low-risk surgeries (e.g., mastectomy) and more stringent for high-risk surgeries (e.g., phalloplasty*).* The BMI criterion leads to moral and ethical challenges for example when used to determine eligibility for preceding treatment steps such as cross-sex hormones (where less is known about medical risks related to BMI). Quoting a psychologist during a team meeting: “He has been trying to lose weight, but unsuccessfully, so it’s very complicated. Are we going to start [hormone] treatment?” Here, the psychologist seemed unsure whether a high BMI is a morally and scientifically permissible exclusion factor for hormone treatment. Implicit moral questions the researchers identified here were: On the basis of what (scientific, experiential) knowledge may a health determinant become an exclusion factor? Who should decide whether the increased risk of a treatment step, plausibly expected because of a specific health determinant, is an exclusion factor?

### Content of Treatment and Care

The moral and ethical dilemmas in this theme revolved around the nature, aspects and experience of individual treatment steps and deals with the (1) medical trajectory, specifically variations from the standard, and (2) (scientific) evidence and uncertainties. The overarching moral questions were: How far can we go with an individually adapted care plan? How should we deal with scientific evidence and uncertainties? What risks may be carried by a transgender individual in treatment, and against what risks should the clinicians protect them?

#### Which Variations from a “Complete” Transgender-Affirming Medical Treatment Should Be Permissible?

The content of a “complete” transgender-affirming medical treatment roughly consists of puberty suppression (in adolescents), hormonal and surgical treatment. However, some only indicate need for individual parts of this trajectory. Often, those individual treatment wishes do not align with the biological and morphological male/female dichotomy, and some of these requests gave rise to moral and ethical challenges. For example, at a team meeting a member rose the question whether a transman who requested mastectomy without having gone through cross-hormonal treatment should be eligible for treatment, for he might have not fully experienced what living as a man entailed. More implicitly, the discussions and notions regarding individual treatment requests elicited implicit questions on the basis of what normative (societal, institutional) presumptions some of these requests were deemed acceptable, while others were not.

#### What Are Appropriate Implications of Current (Scientific) Evidence and Uncertainties?

Moral and ethical challenges regarding (scientific) evidence and uncertainties were plentiful. To illustrate, during a MCD, it appeared unclear to the team members what the evidence for a BMI criterion for hormonal treatment is. A surgeon stated: “The difficult thing is that I cannot put the complication risk into a percentage.” (transcript MCD) And, in another team meeting, a pediatrician asked: “How strict should we be regarding the BMI-criterion for hormone treatment?” These comments raised the question whether a BMI criterion may be used for hormonal treatment, i.e., before any surgical intervention is decided upon. Implicit moral challenges we identified here were: How should caretakers extrapolate general (scientific) evidence, e.g., regarding risk factors and complications, to procedures specific to transgender-affirming medical care? Who should determine what counts as valid evidence? What level of evidence ought there to be for a certain medical criterion to be valid? And, in general, how should caretakers cope with ambiguities in scientific evidence?

### Sequential Order of the Various Treatment Steps

Although the individual treatment steps are regarded as separate entities, in clinical practice they appeared to be sequential and interconnected. For example, hormone treatment functions as a prerequisite for the surgical procedure and if surgery is desired without hormones the local guidelines require the transgender person to have a “thinking” period that is the same amount of time as the hormonal phase would have been. The interconnectedness of the various treatment steps gave rise to moral and ethical difficulties, for example through the dependency on other disciplines for expertise. We identified two subthemes: (1) the multidisciplinary aspect and character of transgender care and (2) the sequential order of the treatment steps. The overarching moral question was: To what extent and in which way should the sequential order and interconnectedness of different parts of transgender care play a role in the individual treatment steps?

#### Should a Lack of Multidisciplinary Consensus Lead to Taking a Step Back in Treatment?

At the CEGD, although intricately connected, a distinction is made between medical and psychological aspects of transgender care, leading to moral and ethical questions about how multidisciplinary consensus and decisions should be reached. For example, in a report of a MCD, a particular case dealt with a trans individual who requested surgery without a social transition due to an unaccepting environment. In this MCD, we noted how participants found it important that multidisciplinary consensus regarding the treatment decision was reached within the team, and that “it can be important to take a step back in order to retain consensus.” Here, we identified the implicit ethical challenges of determining when and why multidisciplinary consensus may necessitate taking a step back. More generally, what should the moral leverage of each discipline be in reaching treatment decisions?

#### Should All Treatment Steps Already Be Taken into Account at the Start?

Some clinicians struggled to decide whether the whole trajectory ought to be considered already in the beginning when determining eligibility for a first individual treatment step. In a MCD, we observed a clinician using the BMI criterion for surgery to establish eligibility for preceding treatment steps such as hormone treatment: “suppose that due to his BMI, he will never get to the surgeries, there is a risk that someone will become even unhappier, by being in a masculinized body with breasts.” (transcript MCD) Here, the clinician is concerned that if you allow a client to start a treatment step now, while being convinced that with the current BMI one will later on not be eligible for surgery, the client may be disappointed and suffer negative sequelae. This evokes the ethical question whether a criterion for treatment B may also hold for treatment A. A normative argument in favor for this was to prevent false expectations: “it could give someone the idea that he’s in the trajectory on his way to masculinize whereas [with this BMI] he will potentially never be eligible for surgery.” (transcript MCD) Weighing these arguments against the potential alleviation hormonal treatment on its own offers, however, is challenging. It is problematized further by uncertainties in evidence for a BMI criterion for the hormonal treatment. More generally, these fragments illustrate the way caretakers explicitly take (potential) future treatment steps into consideration and implicitly consider the multidisciplinary nature of GD treatment in determining eligibility to individual treatment steps.

### The Role of Clinical Guidelines

The clinical guidelines used at the CEGD concurrently provide guidance and give rise to moral and ethical challenges, particularly arising in the context of cases diverging from the guidelines due to a wide variety of (contextual) characteristics. We identified the following subthemes: (1) biological and calendar age and (2) flexibility and strictness of the guidelines. The main ethical question is: Since the guidelines are described as being flexible, and treatment choices that diverge from the guidelines may be made in individual cases, what exemptions are permissible?

#### Should We Go by Biological or Calendar Age?

In children and adolescents’ care, the use of calendar age to determine eligibility for the various treatment steps appeared challenging. According to the local guidelines, in principle adolescents needed to be 12 years old and in pubertal Tanner stage 2–3 to be eligible for hormonal puberty suppression. However, as some reach puberty earlier, a case-by case analysis is opted for in those under 12 to start puberty suppression. The use of calendar age led to an exemplary moral dilemma in a 10.5-year-old girl with early onset of puberty, as according to the psychologist in a team meeting: “[s]he’s too old for the children’s guidelines but too young for the adolescent’s guidelines.” In this case, it was unclear whether the biological age and need for treatment to prevent the development of secondary sex characteristics were outweighed by her limited cognitive abilities to oversee the consequences and precedent. As such, it illustrates the difficulties involved in the balancing act between the guidelines, their flexibility and individual treatment needs.

#### How Strict are the Clinical Guidelines?

We also identified moral and ethical challenges relating to the strictness or lenience with which the clinical guidelines should be interpreted, and to what extent contextual factors may influence this balance. This was a point of frequent discussion between clinicians, for example regarding a case where confusion arose as to whether smoking marijuana should be seen as a firm exclusion criterion for surgery:

Is it bothering you that the guidelines on this topic aren’t clear? I think they shouldn’t be too strict, because every client is different. … There are always particularly upsetting cases where you think: what a sad story. There are just so many circumstances why a person might use these substances. (transcript MCD)

The fragments above show that some team members stress the need for ambiguity in the content or interpretation of the guidelines in striving toward providing good care and illustrate that a more contextual and individual interpretation of the guidelines can be seen as more appropriate when striving toward providing good care. Other team members, however, deem the content or interpretation of the guidelines ambiguous and call for clarifications and a stricter usage: “Well, for me personally, it would be pleasant if we’d be a bit more unambiguous about these guidelines [i.e., on smoking marijuana as an exclusion criterion for surgery].” (transcript MCD) Altogether, these quotes question the aim, status and ultimately the efficiency of the guidelines (i.e., the moral limits of a flexible use of the guidelines*).* We distilled the following moral questions from the quotes above: When is following a guideline becoming an end in itself (instead of delivering good care)? When is it permissible to offer dissimilar interpretations of the same guidelines? When is it right to deviate from the guidelines?

### Notions Regarding Gender Identity and Assessing GD

In some cases, assessing the presence of GD turned out to be a challenging clinical endeavor. Particularly the strategies used to establish the presence of GD and uncertainties regarding its determinability led to challenges. We identified two subthemes: (1) assessing GD and (2) questions regarding the determinability of GD feelings. The main moral questions were: What is the normative status of advice regarding gender expression? And, how should doubt regarding GD and its determinability be dealt with?

#### What Strategies Should Be Used to Diagnose Gender Dysphoria?

Caretakers opted for various strategies to clinically assess the presence of GD (i.e., whether GD was persistent, rather than sudden-onset or short-lived) in order to determine eligibility for treatment, which also includes a diagnostic procedure of secondary importance. For example, when transgender clients are hampered in their ability to express their experienced gender (i.e., verbally, in their demeanor and/or presentation) due to barriers relating to personal resiliency, the procedure of determining eligibility for treatment is hampered. Indeed, when clients were not able to communicate their feelings, desires, convictions and experiences of distress, clinicians found themselves at a loss as to how to proceed in diagnosing and assessing eligibility for medical treatment. These clients may be referred to a specialist for (psychological) training in order to enhance their resiliency, allowing them to express their experienced gender more fully. Sometimes, however, this approach led to moral challenges. During a team meeting a case was discussed in which a transman who appeared to suffer from gender dysphoria neither dared to socially transition nor was open to receiving psychological care. The clinician shared her hesitations to diagnose and to consider this person eligible for transgender-affirming medical treatment. In response, another clinician asked more generally: “how should we actually deal with these kinds of vulnerabilities?”

Similar questions arose in a MCD where a client disregarded the advice to seek psychological assistance in the context of resiliency and gender expression. During this MCD, a clinician shared: “You can always tell people that they should explore [their gender expression] further, but if they don’t, how forceful can that advice be?” This question evoked the following clarifying questions from another clinician: “What is actually our role as advisers? When people fail to follow up on our advices, and we don’t draw consequences, then what are we doing?” (transcript MCD) These fragments indicate that the normative status of advice pertaining to resiliency in the context of gender exploration or expression is unclear, and beg the moral question what the consequences should be when such advice is not followed. A more implicit moral question identified by the researchers here was: Is expressing one’s experienced gender an essential and obligatory condition before one may be diagnosed and medically treated?

#### How Should We Deal with Questions Relating to Determinability?

As some treatment steps are invasive, and (partly) irreversible, the clinicians we observed wanted to make sure that the gender dysphoric feelings were determinable so that the chances of regret would be slim. However, this endeavor led to moral challenges, especially in prepubescent children. A case we observed during a MCD dealt with the potentially overpowering influence of parents and contextual factors: “It was put on this child from the age of three onwards … which makes me worried because I doubt whether this is truly the calling of the child.” (Transcript MCD) In this fragment, the clinician appeared to be hesitant to diagnose, for she was unsure whether the GD feelings were accurately determinable: “He told me memories of early childhood that I knew were not the child’s own, but instigated by the mother. … Then you’re dealing with a complex case.” (transcript MCD) Here, the clinician was struggling to distinguish between the influence of the parents and the genuine feelings of the child, in effect complicating the decision whether to start puberty suppression.

Relatedly, caretakers appeared to assess determinability by considering the temporality with which clients experienced their transgender feelings and expressions, in which the gender dysphoria of those clients presenting with a persistent, life-long or “early-onset” narrative was deemed to be more accurately determinable. However, the usage of this “early-onset narrative” as a means to assess determinability was not without its moral intricacies, for sometimes caretakers feared that, quoting a psychologist at a team meeting: “maybe this story is being told, because [one] thinks it increased [one’s] chance of getting treatment.”

### Decision-Making Process and the Roles of the Stakeholders

As a final theme, we present moral and ethical challenges related to the roles, characteristics and values of the various stakeholders in the decision-making process. We identified two main categories: (1) parents/caretakers and (2) the role of the clinician. The central question is: What is the right balance between protecting transgender individuals and promoting their autonomy?

#### What Role May Parents/Caretakers Have in Reaching Treatment Decisions?

To the clinicians, the role of parents in diagnosis and treatment was vital. According to Dutch medical decision-making law, parents carry full responsibility for children under the age of 12 and shared responsibility with adolescents between 12 and 16 years old for signing informed consent. Socially, caretakers have to be able to provide a stable support system. Contextual factors influencing these legal and social roles lead to moral challenges for caretakers. We observed a discussion regarding how children’s loyalty to their parents may cause them to drop out of treatment. During a team meeting, a clinician said:

It’s a case where parentification due to a troubled past manifests in socially desirable behavior: “When my parents think it’s good for me, then I’ll do that.” As her father did not believe the diagnosis, the adolescent was unsure whether to continue treatment. (team meeting)

This case was complicated further by the fact that the child was 16 years old, and therefore legally allowed to continue treatment without parental support. However, in doing so she would risk a deterioration of her home situation, potentially leading to the absence of parental support for treatment which is a criterion for treatment. In this situation, the clinician found herself morally weighing the adolescent’s parental relationship and support against the potential benefits of transgender-affirming medical treatment.

#### What Should the Role of the Clinician Be in the Decision-Making Process?

Often, clinicians were trapped in a double bind between a protective role on the one hand and an autonomy-promoting role on the other. Determining to what extent the clinician should take responsibility for treatment decisions was often ambiguous. The transgender population is very heterogeneous and hence, defining the boundaries of the protective role hinged on contextual factors:

[T]o what extent is it our responsibility to take a decision regarding these risks? Is that your role as a caretaker, doctor or treating psychologist? Or do you advise someone the best you can about the risks and ask whether the client is willing to take on the responsibility? (transcript MCD)

This fragment indicates that this clinician was experiencing difficulty in defining the limits of professional responsibility.

Relatedly, weighing the risks involved with a certain treatment step against its potential benefits also leads to moral challenges in the context of the client–physician relationship. As told by a clinician after a MCD:

She suddenly started crying and said: “My depression is connected to my gender dysphoria and whether or not I’ll be able to get treatment for it, but I’m afraid to show it [i.e. her depression], as I fear that it’ll be interpreted as a co-morbidity.” (MCD report)

This quote illustrates the moral predicament both transgender individual and clinician may find themselves in. As the former, in fear of being rendered ineligible for treatment, does not feel safe to share the whole story, the latter could be hampered in making the right treatment decision. To ascertain whether the moral and ethical challenges outlined in the findings section resonated with the CEGD’s team members, we conducted a member check which we will elaborate on below.

### Findings from the Member Check with Participating Clinicians

A presentation and discussion of a synthesis of the data took place as a member check. CEGD’s team members were asked whether they recognized the moral and ethical challenges we identified. A psychologist mentioned the following: “yes, I recognize these moral dilemmas, and if you present them like this, I suddenly realize how many decisions we’re making for people.” Another psychologist added: “I was also struck by the sheer amount of problems we face. That there are so many moments in which you can turn either left or right, where apparently I choose to turn left.” One surgeon shared: “I do [recognize these moral and ethical challenges], but this is a markedly different list than I would come up with. I would frame it more in medical situations, or cases about themes such as: children, adults, and surgery.” These responses show caretakers’ recognition of the moral challenges and illustrate how making the latter more explicit, by focusing on the moral and ethical rather than medical content of these challenges, can provide distinctive insights.

Regarding specific themes, a surgeon responded: “We definitely recognize [the problems pertaining to] BMI and smoking.” Another surgeon spoke of the implicit moral challenges concerning individual treatment requests and elaborated: “Not only socially, but also within our team it is difficult to determine what is acceptable,” to which an endocrinologist added, hinting at the challenges working in a multidisciplinary context entail: “Yes, probably because only later [in your career] you start to look beyond the bounds of your discipline. I remember how at first I was only prescribing hormones without taking too much notice of the full trajectory.” Furthermore, the implicit tension between protecting individuals and promoting autonomy was recognized by a psychologist: “It’s interesting to see to what extent we are in control of whether someone can make a next step in the trajectory, and that we decide on that relatively quickly. Looking at it like this makes me realize that we carry an enormous responsibility for the way someone is able to express themselves.” He added: “I thought I knew what a full coming-out entailed, but apparently I have all kinds of presumptions.”

## Discussion

This qualitative ethnographic study investigated the moral and ethical challenges in diagnosing and treating gender dysphoria as experienced by healthcare professionals of a multidisciplinary transgender care team in daily practice. We found that professionals face moral and ethical challenges in (1) determining the circumstances under which transgender individuals should be rendered eligible for treatment; (2) in shaping the content of treatment in the absence of evidence-based consensus and in the context of selective treatment requests; (3) in dealing with the multidisciplinary nature and sequential order of treatment; (4) in establishing the strictness of and possible variations from the clinical guidelines; (5) in assessing the presence and determinability of GD; and finally (6) in the balancing act between protecting transgender clients and promoting autonomy. We will delve into certain salient themes to further illustrate our clinical and embedded findings.

### Are Guidelines Guiding or Prescribing? The Normative Status of the Local Guidelines

Moral and ethical challenges and confusion regarding both the content of the guidelines and the level of flexibility with which they may be handled were abundant. In these discussions, fear of precedent and values such as “justice” in the sense of treating everyone equally resulted in the local guidelines being experienced as a norm: We have guidelines, and we should keep to what’s in them. As such, there appeared to be a continuous tension for caretakers to determine the extent to which the guidelines are guiding or prescribing.

We hypothesize that this phenomenon could in part be due to the specific historical, cultural and legal background in which the local guidelines were developed. For example, until July 2014 in the Netherlands sterilization was a prerequisite to have one’s legal gender recognized by law (art. 1:28 subsection 1 DCC Jo art. 1:20 subsection 1 DCC). This legal requirement was reflected in the local guidelines of that time (Cohen-Kettenis & Gooren, 1999; Delemarre-van de Waal & Cohen-Kettenis, 2006) and hence up until recently, requests for variations from a “complete sexual reassignment surgery” were a priori rejected. Although this legal requirement has subsided, this historical–legal context appears to reverberate in normative arguments issued by some clinicians and the way the local guidelines are effected in practice.

Next, we noticed that guidelines can improve the quality and consistency of clinical decisions, but sometimes at the cost of allowing insufficient elbow room for individuals wishes, circumstances and needs (Woolf, Grol, Hutchinson, Eccles, & Grimshaw, 1999). Moreover, our findings illustrate that the usage of rigid numbers (e.g., BMI as a fixed border; age as an eligibility criterion for puberty suppression) can at once pragmatically enable, but also restrict clinicians in handling the complexity of clinical practice (Woolf et al., 1999). These borders, too, appeared to be associated with Dutch legislation in that, for example, different stakeholders are included in the informed consent procedure regarding treatment decisions involving those above and below the age of 16 (art. 450 subsection 1/2 WGBO). Indeed, compared to the WPATH SOC, some eligibility criteria (e.g., age and BMI) put forward in the local guidelines used at the CEGD appear to be more grounded in values such as “protection” and “good care”.

In order to further contextualize the above, it would be insightful to compare and contrast the moral and ethical challenges we described in relation to the local guidelines to those encountered by other international multidisciplinary transgender teams. However, ways in which such a comparison can aid in answering the question what way of offering care is morally or ethically right should be attenuated for the fact that (a) most commonly used international guidelines are based on expert opinion (Coleman et al., 2012; Hembree et al., 2017); (b) strong empirical data and long-term research supporting particular practices are missing and the quality of research evidence of current empirical data is generally low (Deutsch, Radix, & Reisner, 2016; Feldman et al., 2016); and (c) there are divergent cultural norms in regard to respecting client autonomy and differing health system infrastructures and sociocultural factors affecting the provision of care (Wylie et al., 2016).

### The Fluidity of (Gender) Norms in Gender Care

In transgender-affirming medical care many gender norms are fluid, but so are the norms upheld by clinicians. We found that clinicians consider gender dysphoric feelings more accurately determinable when GD can be rendered early onset (i.e., before puberty), when one is compliant with treatment and has an unambiguous gender presentation, and when role models of both the natal and experienced gender are present during childhood and adolescence. These are exemplary gender norms that influence clinicians in determining eligibility for treatment.

The “early-onset narrative” (Nieder et al., 2011) is a colloquial set of behavioral indicators locating the etiology of gender dysphoria in (early) childhood, implicating a stable transgender identity and focusing on the child’s early response to their natal genitals and typical gender play behavior. Kreukels and Cohen-Kettenis (2011) corroborate the clinical usage of the early-onset narrative as a clinical tool to filter those who benefit from or potentially regret transgender-affirming medical treatment. It is recognized that those suffering from late-onset GD experience greater psychological consequences and higher rates of regret after transgender-affirming surgeries (Zucker, Lawrence, & Kreukels, 2016). Indeed, we found that clinicians’ fear of regret was pervasive, with values such as “well-being” and “security” underlying, e.g., the norm that “regret should be prevented”. However, the use of the “early-onset story” was not without its moral and ethical challenges: In the literature and clinical practice the demarcation between “early” and “late”-onset GD appeared ambiguous: “What [should] count as early onset?” (Zucker et al., 2016, p. 219). Moreover, the influence of potential recall bias, parents/caretakers or anxiety of being rendered ineligible proved to problematize its clinical use further.

Finally, and also regarding the other gender norms mentioned above, our findings show that many contextual factors come into play in shaping and perceiving one’s gender identity, presentation, resilience and commitment to treatment. It is interesting to observe that team members appear to hold various normative presuppositions regarding notions of resilience, commitment and (gender) presentation in the population they are working with. Indeed, as noted by Tishelman et al. (2015), some may not have access or appear committed “because of geography, lack of financial means, and/or because of social structures that do not support them” (p. 42). Hence, deriving normatively laden indicators of (in)eligibility from these (gender) norms can be precarious.

### Moral Challenges of Multidisciplinary and Interdependent Cooperation

In versions preceding the 7th edition of the SoC, the medical path to transitioning was dubbed triadic therapy consisting of a “real-life experience”, hormone treatment and surgery successively. Up until recently, transgender-affirming medical treatment was inherently sequential and binary (Beek, Kreukels, Cohen-Kettenis, & Steensma, 2015). At the CEGD, the sequential order of treatment raised explicit challenges considering varying eligibility criteria (e.g., BMI) and implicit moral questions regarding the eligibility of those seeking individual treatment steps.

Regarding the latter, Kuyper and Wijsen (2014) have sought to quantitatively elucidate various aspects of gender dysphoria in a self-report study among the general (adult) population in the Netherlands. They found that “there is not a one-to-one relationship between gender incongruent feelings, a dislike of one’s natal sex characteristics, and the wish to obtain [full surgical treatment]” (p. 384). Their conclusions empirically support what Cohen-Kettenis and Pfäfflin (2010) dub a “dimensional” over a dichotomous conceptualization of and approach to gender identities, roles and problems.

It is increasingly recognized that the triadic, or sequential model “no longer represents the standard of care” (Wylie et al., 2016). However, our findings attenuate claims such as that from the 6th version of WPATH’s SoC onwards “hormone therapy and surgery are seen as separate treatment options in their own right” (Cohen-Kettenis & Pfäfflin, 2010, p. 503). We illustrated that while some flexible and individualized treatment trajectories were deemed acceptable and devised, others were not. Indeed, individualizing treatment appeared to be a two-edged sword: acknowledging that client’s treatment needs and wishes differ entailed sensitivity toward differing contexts and divergent modes of competence and capacity to engage in shared decision making. In making these assessments, clinicians took potential future treatment steps into consideration, drawing from values such as “protection”, “well-being” and “collegiality”.

In seeking to discern the overarching moral dimension of the challenges we described, we found that in many there appeared to be an underlying, implicit tension between these latter values and the value of client autonomy. Indeed, our findings indicate that often clinicians were trapped in a double bind between a protective role on the one hand and an autonomy-promoting role on the other. In practice, this tension leads to a plethora of moral and ethical questions, both implicit (e.g., “should we start treatment when my client does not oversee or cope with the consequences of treatment?”) and more explicit (e.g., “to what extent should it be my responsibility to come to a decision regarding these risks? What should be the boundary of my professional responsibility?”). Hence, an overarching moral question distilled from our findings is: In our decision-making process, how do we elucidate the various and often diverging moral values we encounter in the intricate, chaotic and fluid reality of clinical practice and how should we go about doing justice to those values most at stake?

In their seminal paper, Emmanuel and Emmanuel (1992) distinguish four models of the client–clinician interaction: (1) the paternalistic model; (2) the informative model; (3) the interpretive model; and (4) the deliberative model. What we observed is that at the CEGD, clinicians alternate in terms of decision-making model from the informative and interpretive in care for competent adult clients, to more deliberative and paternalistic in care for children and adolescents, and clients where psychiatric comorbidity or other factors influence one’s capacity to engage in shared decision making. Indeed, many of the challenges we described are related to the character of these latter medical decision-making models and corresponding client–clinician relationships in which the conception of client values and clinician’s obligations and roles are messier and murkier.

We should emphasize, however, that no matter what decision-making model is opted for, clinicians and clients will always be confronted with moral and ethical challenges. Rather than seeking to “solve” or do away with them, it is vital for those involved in transgender-affirming medical care to develop a sensitivity toward these challenges and an ability to discuss them in an open and vulnerable manner; to engage in a structural dialogue that includes service users. After having discussed some central themes in moral challenges we will now derive tentative clinical and methodological implications from our findings.

### Clinical Implications of This Study: Moral Challenges That Need to Be Addressed

We advocate the further development of the local guidelines through fostering a hermeneutic relationship between “the local guidelines as written” and reflections on its content and normative status. Moreover, in line with other specialized centers, we encourage a critical reflection on the use of more informed and potentially more flexible cutoff scores (regarding, e.g., BMI, calendar age) and a moral substantiation of rigid ones to allow for further individualized care (Tishelman et al., 2015). Additionally, creative means to gauge competence should be developed, especially in younger individuals and those suffering from psychiatric comorbidity.

Second, our findings indicate a need for enhanced awareness of moral gender norms in transgender care and a more systematic handling of them. In this light, it is interesting to point out that multidisciplinary transgender care is usually provided by a range of just clinical disciplines (e.g., psychology, endocrinology, psychiatry, surgery). Another way of structurally enhancing awareness of implicit and explicit (gender) norms in transgender care would be to include members with a background in social science and (medical) ethics. In fact, from 2016 onward, the CEGD multidisciplinary transgender team was supported by the medical humanities department of whom two members have joined the team meetings in order to engender reflection and offer ethics support to the rest of the team. Their endeavors and impressions reach beyond the scope of this paper, but have been described in a different publication (Hartman et al., 2018b).

Another way in which the former could be met is by engaging in an ongoing and reciprocal conversation with service users elucidating the moral and ethical challenges encountered when *receiving* medical care, potentially making use of these gender narratives to inform and develop a multidisciplinary (bias) training module on how to engage with the inherent moral dimension of transgender-affirming medical care for caregivers. The latter becomes especially relevant when considering that contextual factors influence one’s gender identity, gender expression, corresponding treatment wishes and ability to commit to treatment. These embedded realities should guide individually catered care. Relatedly, important avenues for future research include the moral and practical implications of a more dimensional approach toward transgender-affirming medical care.

Third, we argue that the sequential steps of care and corresponding eligibility criteria should be contrasted with the particular (selective and individual) treatment wishes envisioned by the client. Elucidating the latter will foster a more explicit moral deliberation in which the potential therapeutic benefits of single treatment steps can be weighed against potential pitfalls. Finally, our findings underscore the call for sound qualitative and quantitative research to inform guidelines and best practices, along with follow-up research on experiences with the provided care (Deutsch et al., 2016; Feldman et al., 2016).

### The Potential Contribution of Clinical Ethics Support in Transgender Care

The clinicians we observed called for means to grapple with moral and ethical challenges. Our findings stress that clinicians in transgender-affirming medical care engage in several balancing acts and tensions, rendering their profession morally and philosophically challenging, which is an argument for more integrated CES in transgender-affirming medical care (Hartman et al., 2018b).

Systematic CES, through offering a more constructive and methodical approach to moral and ethical challenges, can make these normative underpinnings explicit, and, by means of its “outsider perspective”, more transparent. Moreover, CES can foster clinicians’ openness to the contextuality and contingency of the moral challenge they are faced with (Molewijk, Slowther, & Aulisio, 2016), in effect aiding the dynamic process of reaching and substantiating (treatment) decisions. It is important to add here that this process requires CES staff to employ a delicate balance between taking care for the relationship and win the trust of clinicians on the one hand and being critical and explicitly normative on the other. This tension is discussed in another publication (Hartman et al., 2018b).

Finally, the member check of this research illustrates that research in itself can be a tool for CES through fostering awareness and discussion on moral issues that professionals until then were not aware of. As such, the main clinical implication of this research is that transgender-affirming medical care requires continuous moral deliberation and sensitivity toward (normative) intuitions, presuppositions, claims and changing contextual factors. Ongoing moral deliberation on what constitutes good care is in itself an element of (re)constructing good care.

### Strengths and Limitations

The qualitative–observational nature of our research allowed us to add to the current literature in a threefold manner. First, by providing a more detailed insight into the contextuality of moral challenges in clinical practice. Second, by identifying not only explicit but also more implicit moral challenges by using a partly outsider perspective. Third, by elucidating the way professionals *experience* these moral challenges.

There are also limitations to this present study. First, we methodologically focused on the ways in which *clinicians* experience moral challenges. Hence, our methodology did not allow for a rigorous teasing apart of what challenges might be born out of, e.g., clinician bias, derive from the way local standards are devised and worked with; or more generally relate to varying implicit and explicit normative presuppositions team members hold regarding the nature of GD, its population and treatment. It should be noted that our vision on ethics is dialogical: As researchers and ethicists, within the joint research we execute, we are in a continuous dialogue on what constitute good care. Through dialogue a mutual learning process emerges, in which all stakeholders involved critically learn to understand what is conceived as morally good care. It is rather this process of joint critical engagement and reflection through a dialogical learning process than an outsider’s perspective in which clinical practices and the professionals working therein are morally judged.

Furthermore, the hermeneutic theoretical viewpoint on clinical ethics and integrative ethics support emphasizes that differences within moral and ethical challenges will always be there and are seen as good catalyzers of moral learning: People(/clinicians) are never “neutral” and they always have a (dynamic) normative frame of interpretation and reference (Widdershoven & Molewijk, 2010). People hold normative presuppositions, and indeed, these various presuppositions may come into conflict. However, viewed from our theoretical framework, this is not a problem or weakness per se: It is rather seen as the start of a new moral learning (Abma, Molewijk, & Widdershoven, 2009; Widdershoven et al., 2009).

Next, although sufficiently demarcated, the themes described are highly entangled. Some topics such as substance abuse and BMI appeared omnipresent within the various themes. Next, this research relied on CES, particularly MCD, as a data collection method. On the one hand, this specific and methodical focus on the moral dimensions of transgender-affirming medical care has proven to be beneficial in laying bare moral and ethical questions and dilemmas. On the other hand, this enhanced sensitivity is likely to have influenced the multidisciplinary team’s attention toward moral and ethical challenges. Their openness to this attention, however, is laudable and should be considered a strength of this research.

Relatedly, some quotes have been altered slightly or omitted at the request of members of the team without editing or excluding the moral or ethical issue at hand. This made us aware that paying attention to the moral dimension of the clinical work within transgender-affirming medical care and the verbal expressions used therein sensitized team members’ responsiveness toward the moral and ethical intricacies of their profession. As such, integrative ethics support can be seen as an ongoing transformative learning process.
